# Prostate cancer risk and consumption of fish oils: a dietary biomarker-based case–control study

**DOI:** 10.1038/sj.bjc.6690835

**Published:** 1999-12

**Authors:** A E Norrish, C M Skeaff, G L B Arribas, S J Sharpe, R T Jackson

**Affiliations:** Department of Community Health, University of Auckland, PO Box 92-019, Auckland, New Zealand; Department of Human Nutrition, University of Otago, Dunedin, New Zealand; Department of Medicine, University of Auckland, Auckland, New Zealand

**Keywords:** prostatic neoplasms, diet, biomarker, eicosapentaenoic acid, docosahexaenoic acid

## Abstract

Experimental studies suggest that the risk of prostate cancer is reduced with the intake of long-chain n-3 polyunsaturated fatty acids derived from marine foods, such as eicosapentaenoic acid (EPA) and docosahexaenoic acid (DHA). However, few human studies have been conducted due to difficulties in assessing the dietary intake of these fatty acids. The authors examined the relationship between prostate cancer risk and EPA and DHA in erythrocyte biomarkers in a population-based case–control study in Auckland, New Zealand during 1996–1997 involving 317 prostate cancer cases and 480 age-matched community controls. Reduced prostate cancer risk was associated with high erythrocyte phosphatidylcholine levels of EPA (multivariate relative risk = 0.59; 95% confidence interval 0.37–0.95, upper vs lowest quartile) and DHA (multivariate relative risk = 0.62; 95% confidence interval 0.39–0.98, upper vs lowest quartile). These analyses support evidence from in vitro experiments for a reduced risk of prostate cancer associated with dietary fish oils, possibly acting via inhibition of arachidonic acid-derived eicosanoid biosynthesis. © 1999 Cancer Research Campaign


*British Journal of Cancer* (1999) **81**(7), 1238–1242
© 1999 Cancer Research Campaign
Article no. bjoc.1999.0835

### Prostate cancer risk and consumption of fish oils: a dietary biomarker-based case–control study

**AE Norrish[1], CM Skeaff[2], GLB Arribas[2], SJ Sharpe[3] and RT Jackson[1]**

[1]Department of Community Health, University of Auckland, PO Box 92-019, Auckland, New Zealand; [2]Department of Human Nutrition, University of Otago, Dunedin, New Zealand; [3]Department of Medicine, University of Auckland, Auckland, New Zealand

**Summary** Experimental studies suggest that the risk of prostate cancer is reduced with the intake of long-chain n-3 polyunsaturated fatty acids derived from marine foods, such as eicosapentaenoic acid (EPA) and docosahexaenoic acid (DHA). However, few human studies have been conducted due to difficulties in assessing the dietary intake of these fatty acids. The authors examined the relationship between prostate cancer risk and EPA and DHA in erythrocyte biomarkers in a population-based case–control study in Auckland, New Zealand during 1996–1997 involving 317 prostate cancer cases and 480 age-matched community controls. Reduced prostate cancer risk was associated with high erythrocyte phosphatidylcholine levels of EPA (multivariate relative risk = 0.59; 95% confidence interval 0.37–0.95, upper vs lowest quartile) and DHA (multivariate relative risk = 0.62; 95% confidence interval 0.39–0.98, upper vs lowest quartile). These analyses support evidence from in vitro experiments for a reduced risk of prostate cancer associated with dietary fish oils, possibly acting via inhibition of arachidonic acid-derived eicosanoid biosynthesis. © 1999 Cancer Research Campaign

**Keywords:** prostatic neoplasms; diet; biomarker; eicosapentaenoic acid; docosahexaenoic acid

The wide international variation in prostate cancer incidence, and increased risks for men who migrate from low- to high-risk countries, provide indirect evidence for the existence of environmental risk factors for prostate cancer (Nomura and Kolonel, 1991). Dietary factors, particularly the consumption of fat, have been identified as likely environmental risk factors for prostate cancer (Kolonel, 1996; Giovannucci, 1995), but the underlying mechanisms and specific dietary risk factors remain unclear. It has been proposed that through competitive inhibition of cyclooxygenase and lipoxygenase pathways, n-3 polyunsaturated fatty acids from marine foods may modify the biosynthesis of arachidonic acid-derived prostaglandins and other eicosanoids and thereby inhibit prostate carcinogenesis or progression (Karmali, 1987). Experimental studies involving the human prostate cancer cell lines PC-3 and DU145 have shown inhibition of cell proliferation rates with exposure to long-chain n-3 polyunsaturated fatty acids including eicosapentaenoic acid (EPA, C20:5n-3) and docosahexaenoic acid (DHA, C22:6n-3) (Rose, 1997). There have been few human studies of the influence of dietary EPA and DHA on prostate cancer risk since daily intake of these individual fatty acids is relatively low in most Westernized countries, and valid and reliable dietary assessment instruments have not been available with which to estimate their consumption. In order to address these concerns, the current study aimed to investigate associations between prostate cancer risk and EPA and DHA intake assessed primarily by erythrocyte-based biomarker measurements.

*Received 9 February 1999*
*Revised 16 April 1999*
*Accepted 19 April 1999*

*Correspondence to:* AE Norrish

### MATERIALS AND METHODS

### The study participants

The Auckland Prostate Study is a population based case–control study which was carried out in the greater metropolitan area of Auckland, New Zealand. The study population included all men aged 40–80 years normally resident in the Auckland area during the 13-month study recruitment period from January 1996. Almost all men with newly diagnosed prostate cancer in this Auckland population age group attend specialist urologists, either in one public hospital-based clinic, or one of three private clinics involving seven urologists. In this study, all public hospital urology clinic attendees and patients attending five of seven private clinic urologists were eligible for participation. In order to improve response rates and reduce dietary assessment biases arising from knowledge of a recent cancer diagnosis, the majority of prostate cancer cases were recruited prospectively from a larger group of urology clinic attendees prior to the completion of clinic investigations for prostate cancer. The remainder of prostate cancer cases in the study population were identified retrospectively from histology reports, but within 3 weeks of the cancer diagnosis. All prostate cancer cases were confirmed by reference to histology reports. The study identified a sub-group of ‘advanced’ cancer cases who were men with clinically significant disease. The definition of ‘advanced’ cancers was defined prior to study analyses and was based upon tumour stage and grade. It comprised cases with documented pathological or radiological evidence of tumour invasion beyond the prostate capsule, or a tumour with a combined Gleason score of seven or more.

Study controls comprised men aged 40–80 years, with no history of prostate cancer, randomly selected from the general electoral rolls (these provide 95% coverage of adult European men

**Table 1** Characteristics of the study participants – controls and total prostate cancers

| Characteristic | Controls $n$ = 480 | Total cases $n$ = 317 | $P$-value[a] |
|---|---|---|---|
| Age – mean years (s.e.m.) | 69.1 (0.3) | 68.3 (0.4) | 0.10 |
| Socio-economic status – number[b] (%) | | | |
| – high | 211 (44) | 98 (31) | |
| – middle | 114 (24) | 85 (27) | |
| – low | 149 (31) | 129 (41) | 0.001 |
| Non-steroidal anti-inflammatory drug treatment[c] – number (%) | 176 (37) | 105 (33) | 0.27 |
| Total energy intake – mean kJ $day^{-1}$ (s.e.m.) | 8818 (117) | 8929 (158) | 0.57 |
| Total fat intake – mean g $day^{-1}$ (s.e.m.) | 76.2 (1.3) | 77.2 (1.7) | 0.67 |
| Total polyunsaturated fat intake – mean g $day^{-1}$ (s.e.m.) | 9.9 (0.2) | 10.5 (0.3) | 0.12 |
| Total saturated fat intake – mean g $day^{-1}$ (s.e.m.) | 33.3 (0.7) | 33.5 (0.9) | 0.89 |
| Total monounsaturated fat intake – mean g $day^{-1}$ (s.e.m.) | 24.9 (0.5) | 25.0 (0.6) | 0.86 |
| Total eicosapentaenoic acid intake – mean g $day^{-1}$ (s.e.m.)[d] | 0.07 (0.004) | 0.07 (0.004) | 0.59 |
| Total docosahexaenoic acid intake – mean g $day^{-1}$ (s.e.m.)[d] | 0.11 (0.006) | 0.10 (0.006) | 0.65 |
| Lycopene intake – mean µg $day^{-1}$ (s.e.m.) | 1616 (80) | 1428 (69) | 0.08 |
| Erythrocyte eicosapentaenoic acid – mean mol % (s.e.m.) | 0.67 (0.02) | 0.62 (0.02) | 0.05 |
| Erythrocyte docosahexaenoic acid intake – mean mol % (s.e.m.) | 1.47 (0.02) | 1.38 (0.03) | 0.01 |

[a]$P$-value for the comparison of total cases versus controls. [b]Total numbers may be incomplete due to missing data for some observations. [c]Regular use of aspirin or other non-steroidal anti-inflammatory drugs. [d]Estimated daily intake based upon food frequency questionnaire data.

in the Auckland region). Control participants were group-matched to cases during the study recruitment period using 10-year age groups and an approximate case–control ratio of 1:1.5. Approval to carry out the study was obtained from the Northern Regional Health Authority Ethics Committee and written consent was obtained from all participants.

### Collection of questionnaire data

Identical procedures were used for exposure data collection from cases and controls. Study participants completed self-administered questionnaires including a validated 107 food-item food frequency questionnaire (Sharpe et al, 1993) and a general questionnaire covering personal, socio-demographic, anthropometric, medical and lifestyle data. Spearman correlation coefficients for estimated intake of total fat and total polyunsaturated fat (food frequency questionnaire vs 7-day food record) were 0.69 and 0.63 respectively (Sharpe et al, 1993). Research nurses visited all participants at home to obtain blood samples and to check the completeness of responses to the questionnaires which had been posted to participants 1–2 weeks previously. Participants with missing responses were encouraged to fully complete the questionnaires (self-administered) at the time of the visit. Although the nurse-interviewers were not 'blind' to the case–control status of participants, neither they nor the participants were aware of specific hypotheses concerning diet and prostate cancer risk.

### Blood sample processing and erythrocyte fatty acid analysis

Ten millilitres of whole blood was collected from participants in EDTA and transported promptly to the laboratory in chilled polystyrene containers. Erythrocytes were separated from plasma and washed three times in HEPES-buffered saline. Centrifugation of samples was carried out at 4°C. Washed erythrocyte samples were transferred to cryovials (Greiner) allowing minimal headspace air in each vial and stored at –82°C for a period of 3–12 months prior to further analysis. Lipids were extracted from packed erythrocytes according to the method of Bligh and Dyer (1959) and fatty acid composition of erythrocyte phosphatidylcholine was determined using gas-liquid chromatography, as described by Holub and Skeaff (1987). The precision of the fatty acid measurement was determined by repeated analyses ($n$ = 22) of a pooled erythrocyte sample carried out over the analysis period. Coefficients of variation for EPA and DHA were 7.3% and 8.2% respectively. The addition of internal standard to sample and blank extractions permitted sample peak areas to be corrected for background contamination. Quantification of the area under the curves for individual fatty acids allowed calculation of the molar proportion of the total fatty acids. Laboratory staff were kept blind to the case–control status of participants' blood samples.

### Statistical analysis

Nutrient consumption was estimated from the food frequency questionnaire data and New Zealand food composition tables (Burlingame et al, 1993). Categories of exposure for nutrients and the molar percentage of erythrocyte fatty acids were defined by quartiles, based upon the distribution in the control group. Relative risks for total and advanced prostate cancers were calculated for quartile categories, with the reference group comprised of the lowest quartile. Since controls were matched to cases by age group during recruitment, age-adjusted relative risks were calculated for all analyses. Multivariate relative risks were calculated using an unconditional logistic regression model. Age was included as a continuous variable in regression models, but other co-variates were included as categorical terms. Socio-economic status was defined by the participants' usual current or former occupation (if retired), according to the modified Elley-Irving Classification (Johnston, 1983) which has been widely used in New Zealand population research. Energy-adjustment of fatty acid data was carried out by including categorical terms for total energy consumption (derived from the food frequency questionnaire) in the logistic regression model. A test for overall trend across categories used the $P$-values from a logistic regression model, which included ordinal terms for each quartile of intake as well as confounding co-variates.

*© 1999 Cancer Research Campaign*

**Table 2** Prostate cancer relative risk and eicosapentaenoic acid and docosahexaenoic acid levels in erythrocyte phosphatidylcholine, by quartile, total and advanced cancers

| | | Quartile – relative risk (95% confidence interval) | | | | |
|---|---|---|---|---|---|---|
| **Fatty acid** | | **Q1** | **Q2** | **Q3** | **Q4 (high)** | ***P* for trend** |
| Eicosapentaenoic acid (C20:5n-3) | – mol %[a] | < 0.44 | 0.44–0.62 | 0.62–0.83 | > 0.83 | |
| | Total cancers: | | | | | |
| | – age-adjusted RR | 1.00 | 0.65 (0.43–0.99) | 0.70 (0.46–1.05) | 0.60 (0.39–0.92) | 0.02 |
| | – multivariate RR[b] | 1.00 | 0.74 (0.47–1.14) | 0.72 (0.46–1.12) | 0.59 (0.37–0.95) | 0.03 |
| | Advanced cancers: | | | | | |
| | – age-adjusted RR | 1.00 | 0.66 (0.41–1.07) | 0.68 (0.42–1.10) | 0.57 (0.34–0.94) | 0.03 |
| | – multivariate RR[b] | 1.00 | 0.73 (0.43–1.22) | 0.65 (0.38–1.11) | 0.54 (0.31–0.96) | 0.03 |
| Docosahexaenoic acid (C22:6n-3) | – mol %[a] | < 1.13 | 1.13–1.42 | 1.42–1.70 | > 1.70 | |
| | Total cancers: | | | | | |
| | – age-adjusted RR | 1.00 | 0.80 (0.53–1.20) | 0.53 (0.34–0.83) | 0.66 (0.43–1.00) | 0.02 |
| | – multivariate RR[b] | 1.00 | 0.83 (0.53–1.28) | 0.54 (0.33–0.87) | 0.62 (0.39–0.98) | 0.01 |
| | Advanced cancers: | | | | | |
| | – age-adjusted RR | 1.00 | 0.83 (0.52–1.32) | 0.44 (0.26–0.76) | 0.73 (0.45–1.18) | 0.06 |
| | – multivariate RR[b] | 1.00 | 0.84 (0.51–1.39) | 0.46 (0.25–0.82) | 0.66 (0.39–1.13) | 0.04 |

[a]Based upon percentage of total peak areas. [b]Multivariate logistic regression model including terms for age, height, total non-steroidal anti-inflammatory drug use, socio-economic status, and food frequency questionnaire-estimated intake of total polyunsaturated fat and lycopene.

### RESULTS

Participants in the Auckland Prostate Study included a total of 317 prostate cancer cases (77% response rate) and 480 controls (71% response rate). The results of blood sample analyses were available for 285 cases (90%) and 427 (89%) of study controls. Due to the age-matched recruitment process there was little difference in age distribution of cases and controls, however, cases were of lower socio-economic status and less likely to receive regular treatment with non-steroidal anti-inflammatory drugs (Table 1). Energy and total fat intake was similar between the two groups, but cases reported a lower mean intake of lycopene and had lower erythrocyte levels of EPA and DHA. Examination of these characteristics amongst participants for whom blood results were available revealed only very minor differences from the total study population.

For the control group the Spearman coefficient ($r$) for the correlations between erythrocyte phosphatidylcholine EPA measurement and food frequency questionnaire-estimated daily intake of total fish was 0.26; for DHA, Spearman $r = 0.32$. Similar correlation coefficient values were observed for cases. A significant inverse association was observed between erythrocyte measurements of EPA and DHA and the risk of prostate cancer (Table 2). This association persisted following adjustment for a number of potential confounding variables included in a multivariate model. Energy-adjusted analyses resulted in negligible differences in relative risk estimates and are not presented. However, estimates of daily EPA and DHA intake derived from the food frequency questionnaire were not significantly associated with prostate cancer risk; the multivariate relative risk for the upper quartile versus the lowest quartile of EPA intake was 0.96 (95% confidence interval (CI) 0.63–1.48); and for DHA intake the relative risk was 1.10 (95% CI 0.71–1.70).

### DISCUSSION

This population-based case–control study of prostate cancer has observed a reduction in prostate cancer risk associated with dietary biomarker measurements of EPA and DHA intake, but not with estimated dietary intakes of these fatty acids derived from self-reported food consumption.

A major goal of our study was to incorporate more accurate measurements of dietary exposure to fish oils. The molar proportion of individual erythrocyte fatty acids quantified in our study represents a relative measure of fatty acid exposure integrated over time at the circulation level, closer to the target tissue of concern. This approach reduces some of the uncertainties concerning self-reported diets and nutritional database inadequacies. Several intervention studies of human subjects given controlled diets of EPA and DHA (including those with moderate intake levels) have demonstrated clear associations with the erythrocyte fatty acid composition of these fatty acids (Glatz et al, 1989; Agren and Hanninen, 1991; Brown et al, 1991; Vajreswari and Narayanareddy, 1992). Although not directly examined in erythrocytes, it has been demonstrated that the consumption of preformed EPA and DHA in the diet leads to a greater increase of these fatty acids in plasma and platelet phospholipids than an equivalent intake of dietary alpha-linolenic acid (Sanders and Roshanai, 1983; Mantzioris et al, 1994), suggesting that dietary intake, rather than endogenous biosynthesis is the major determinant of circulating levels in humans.

We observed only moderate correlations between erythrocyte levels of EPA and DHA and self-reported fish intake, but similar correlation values existed for prostate cancer cases and controls. These findings are similar to those reported by a previous small case–control study of prostate cancer (Godley et al, 1996*a*). They suggest that the food frequency questionnaires used in these studies may not constitute a very sensitive and accurate means of assessing EPA and DHA intake, particularly in study populations with relatively restricted intakes of marine food sources. However, the similar correlations for prostate cancer cases and controls provide indirect evidence that prostate cancer does not seriously disrupt the relationship between dietary fatty acids and their incorporation into erythrocytes, an important theoretical concern with the use of biological measurements of exposure in case–control studies. Although differences in plasma fatty acids observed

 *© 1999 Cancer Research Campaign*

between colorectal cancer patients and controls have been attributed, at least in part, to metabolic changes associated with malignancy, corresponding differences were not reported for erythrocyte fatty acids in the same study (Baro et al, 1998).

The initial participant response rates are typical of those achieved by this type of case–control study design; however, incomplete recruitment raises the potential for selection bias in our study. The excluded cases attending two out of seven private urologists were estimated to represent only 9% of the total number of cases, and were expected to be representative of private cases overall. While their exclusion may be expected to contribute to differences in the socio-economic status of cases and controls, all analyses were adjusted for this variable. By comparing the characteristics of participants with available and missing erythrocyte data, we ascertained that the availability of blood sample analyses was unrelated to the exposure or disease status of participants, and unlikely to result in selection bias. Since the majority of cases in our study (60%) provided blood samples and questionnaire data prior to their cancer diagnosis, or shortly after diagnosis in the remainder (retrospectively recruited cases), the potential for bias involving dietary recall or modification may be less than in traditional case–control study designs.

Few previous human prostate cancer studies have investigated associations with marine foods or longer chain n-3 polyunsaturated fatty acids due to dietary assessment limitations. Two case–control studies involving Japanese and British men have suggested that prostate cancer risk may be raised amongst men who reported consuming little seafood in their diet (Mishina et al, 1985; Ewings and Bowie, 1996), but a cohort study conducted in the USA observed no association with long-chain n-3 fat from fish (Giovannucci et al, 1992), a result consistent with our own food frequency questionnaire-derived findings. Only three previous studies have incorporated dietary biomarkers. A small urology clinic-based case–control study involving 89 cases and 38 controls has reported a reduced prostate cancer risk associated with the highest quartile of EPA and DHA levels in erythrocytes, but the precision of relative risk estimates was low and there was no clear gradient of risk over increasing levels of exposure (Godley et al, 1996*b*). No significant associations with EPA or DHA were noted by two small prospective studies involving 120 cases and 35 cases respectively (Gann et al, 1994; Harvei et al, 1997), which measured fatty acids in stored serum and plasma samples collected as part of earlier studies (these samples may provide a less valid biomarker measurement of dietary fatty acid intake compared with erythrocyte or adipose tissue samples collected specifically for this purpose).

The underlying cellular and molecular processes by which fish oils may reduce prostate cancer risk have not been established; however, mechanisms involving the inhibition of cancer progression via modulation of arachidonic acid-derived eicosanoid biosynthesis are plausible (Earnest et al, 1992; Lupulescu, 1996). EPA and DHA have been shown to inhibit the augmented production of $PGE_2$ by malignant prostate tissue (Chaudry et al, 1994) and cell proliferation rates in human prostate cancer cell lines in vitro (Karmali, 1987; Rose and Connolly, 1991), although a more recent experimental study demonstrated an inhibitory effect of EPA only at high doses (Pandalai et al, 1996). Many of these experimental studies have also demonstrated inhibition of prostate cancer cell proliferation with non-steroidal anti-inflammatory drugs (cyclooxygenase inhibitors) and a reduced risk of advanced prostate cancer has been previously reported amongst men in our study who regularly took non-steroidal anti-inflammatory drugs (Norrish et al, 1998).

In conclusion, our observations of a reduced prostate cancer risk associated with increased levels of long-chain n-3 polyunsaturated fatty acids in erythrocytes are supportive of eicosanoid-based hypotheses for prostate carcinogenesis or progression, and warrant further investigation.

### ACKNOWLEDGEMENTS

The authors would like to acknowledge the provision of funding for this research from the Cancer Society of New Zealand and the Health Research Council of New Zealand, the assistance with patient recruitment provided by Mr Colin McRae and his colleagues in the Department of Urology, Auckland Hospital, and the processing and transportation of blood samples by Kathy Figgans and staff of the Auckland Regional Blood Centre. Special acknowledgment is made of technical assistance given by Cherie Mulholland, Susan Hawkins and Fleur O'Keefe.

### REFERENCES

Agren JJ and Hanninen OO (1991) Effect of moderate freshwater fish diet on erythrocyte ghost phospholipid fatty acids. *Ann Med* **23**: 261–263

Baro L, Hermoso J-C, Nunez M-C, Jiminenez-Rios J-A and Gil A (1998) Abnormalities in plasma and red blood cell fatty acid profiles of patients with colorectal cancer. *Br J Cancer* **77**: 1978–1983

Bligh DG and Dyer WJ (1959) A rapid method of total lipid extraction and purification. *Can J Biochem Physiol* **37**: 911–917

Brown AJ, Pang E and Roberts DC (1991) Persistent changes in the fatty acid composition of erythrocyte membrance after moderate intake of n-3 polyunsaturated fatty acids: study design implications. *Am J Clin Nutr* **54**: 668–673

Burlingame BA, Milligan GC, Apimerika DE and Arthur JM (1993) *The Concise New Zealand Food Composition Tables*. Palmerston North, NZ: New Zealand Institute for Crop and Food Research Limited, Department of Health

Chaudry AA, Wahle KWJ, McClinton S and Moffat LEP (1994) Arachidonic acid metabolism in benign and malignant prostatic tissue in vitro: effects of fatty acids and cyclooxygenase inhibitors. *Int J Cancer* **57**: 176–180

Earnest DL, Hixson LJ and Alberts DS (1992) Piroxicam and other cyclooxygenase inhibitors: potential for cancer chemoprevention. *J Cell Biochem* **161** (suppl): 156–166

Ewings P and Bowie C (1996) A case–control study of cancer of the prostate in Somerset and East Devon. *Br J Cancer* **74**: 661–666

Gann PH, Hennekens FM, Sacks FG, Grodstein F, Giovannucci EL and Stampfer MJ (1994) Prospective study of plasma fatty acids and risk of prostate cancer. *J Natl Cancer Inst* **86**: 281–286

Giovannucci E (1995) Epidemiological characteristics of prostate cancer. *Cancer* **75**: 1766–1777

Giovannucci E, Rimm EB, Colditz GA, Stampfer MJ, Asherio A, Chute CC and Willett WC (1992) A prospective study of dietary fat and risk of prostate cancer. *J Natl Cancer Inst* **85**: 1571–1579

Glatz JFC, Soffers AE and Katan MB (1989) Fatty acid composition of serum cholesterol esters and erythrocyte membranes as indicators of linoleic acid intake in man. *Am J Clin Nutr* **49**: 269–276

Godley PA, Campbell MK, Miller C, Gallagher P, Martinson FE, Mohler JL and Sandler RS (1996*a*) Correlation between biomarkers of omega-3 fatty acid consumption and questionnaire data in African American and Caucasian United States males with and without prostatic carcinoma. *Cancer Epidemiol Biomarkers Prev* **5**: 115–119

Godley PA, Campbell MK, Gallagher P, Martinson FEA, Mohler JL and Sandler RS (1996*b*) Biomarkers of essential fatty acid consumption and risk of prostatic carcinoma. *Cancer Epidemiol Biomarkers Prev* **5**: 889–895

Harvei S, Bjerve KS, Tretli S, Jellum E, Robsahm TE and Vatten L (1997) Prediagnostic level of fatty acids in serum phospholipids: Ω-3 and Ω-6 fatty acids and the risk of prostate cancer. *Int J Cancer* **71**: 545–551

Holub BJ and Skeaff CM (1987) Nutritional regulation of cellular phosphatidylinositol. *Methods Enzymol* **141**: 234–244

© 1999 Cancer Research Campaign

Johnston R (1983) *A Revision of Socio-economic Indices for New Zealand*. New Zealand Council for Educational Research: Wellington
Karmali RA (1987) Fatty acids: inhibition. *Am J Clin Nutr* **45**: 225–229
Kolonel LN (1996) Nutrition and prostate cancer. *Cancer Causes Control* **7**: 83–94
Lupulescu A (1996) Prostaglandins, their inhibitors and cancer. *Prostaglandins Leuko Essent Fatty Acids* **54**: 83–94
Mishina T, Watanabe H, Araki H and Nakao M (1985) Epidemiological study of prostatic cancer by matched pair analysis. *Prostate* **6**: 423–436
Mantzioris E, James MJ, Gibson RA and Cleland LG (1994) Dietary substitution with an alpha-linolenic acid-rich vegetable oil increases eicosapentaenoic acid concentrations in tissues. *Am J Clin Nutr* **59**: 1304–1309
Nomura AM and Kolonel LN (1991) Prostate cancer: a current perspective. *Am J Epidemiol* **139**: 200–227
Norrish AE, Jackson RT and McRae CU (1998) Non-steroidal anti-inflammatory drugs and prostate cancer progression. *Int J Cancer* **77**: 511–515
Pandalai PK, Pilat MJ, Yamazaki K, Naik H and Pienta KJ (1996) The effects of omega-3 and omega-6 fatty acids on in vitro prostate cancer growth. *Anticancer Res* **16**: 815–820
Rose DP (1997) Effects of dietary fatty acids on breast and prostate cancers: evidence from in vitro experiments and animal studies. *Am J Clin Nutr* **66** (suppl): 1513S–1522S
Rose DP and Connolly JM (1991) Effects of fatty acids and eicosanoid synthesis on the growth of two prostate cancer cell lines. *Prostate* **18**: 243–254
Sanders TA and Roshanai F (1983) The influence of different types of omega 3 polyunsaturated fatty acids on blood lipids and platelet function in healthy volunteers. *Clinical Science* **64**: 91–99
Sharpe SJ, Page NA, Gamble GD and Sharpe CM (1993) Validation of a food frequency questionnaire for use in cardiovascular risk factor studies in New Zealand. *Proc Nut Soc NZ* **18**: 90–100
Vajreswari A and Narayanareddy K (1992) Effect of dietary fats on erythrocyte membrane lipid composition and membrane-bound enzyme activities. *Metabolism* **41**: 352–358

*© 1999 Cancer Research Campaign*
